# Characteristics of Wheat Noodle “Kitanokaori” Using Weakly Acidic Hard Water in Terms of Functional Qualities, Such as Inhibiting Postprandial Abrupt Increase in Blood Glucose

**DOI:** 10.3390/foods14061044

**Published:** 2025-03-19

**Authors:** Sumiko Nakamura, Ken’ichi Ohtsubo

**Affiliations:** Faculty of Applied Life Sciences, Niigata University of Pharmacy and Applied Life Sciences, 265-1, Higashijima, Akiha-ku, Niigata 956-8603, Japan; snaka@nupals.ac.jp

**Keywords:** noodle, calcium, hard wheat, soft wheat, weakly acidic hard water

## Abstract

Type 2 diabetes and osteoporosis are very serious diseases all over the world. We prepared noodles from ‘Kitanokaori’ (newly developed wheat) (KITs) using weakly acidic hard water, which showed a higher amount of resistant starch (9.0-fold) and calcium (2.7-fold) than noodles from Sanukinoyume (premium wheat) (SANs) using purified water. Furthermore, aged mice, which were fed a diet of KIT using weakly acidic hard water for eight weeks, showed lower postprandial blood glucose levels (BGLs) at 30 min after consumption than mice fed a control diet (SAN using purified water) (*p* < 0.05). Therefore, KIT seems promising in terms of health promotion through food. Additionally, the whiteness (WB) and brightness (L*) of wheat noodles using weakly acidic hard water showed higher values than ones using purified water. The texture of KIT using weakly acidic hard water showed few textural differences from noodles using purified water. The KIT using weakly acidic hard water would be acceptable in terms of palatability and bio-functionality in terms of delaying digestion.

## 1. Introduction

With the increase in aged people, prevention of lifestyle-related diseases has become more important year by year all over the world. For example, 537 million people were estimated to be suffering from diabetes [1], and the number of patients suffering from dementia was about 57 million in the world [2]. Furthermore, people with diabetes seem to develop dementia with two times higher probability than healthy people [3].

In addition to the recommendation of low glycemic index (GI) foods by WHO and FAO, it was reported that diabetes and cognitive decline can be prevented by bio-functional food ingredients, such as omega-3 PUFAs (polyunsaturated fatty acids), polyphenols, flavonoids, various minerals, etc. [4,5,6,7,8].

Diabetes is more common in the Western Pacific Region because South Asian people’s bodies do not produce as much insulin as other people. There are many reports that calcium is an essential mineral for humans; nevertheless, dietary intake as calcium is insufficient for preventing diabetes among Asian people [9,10].

Wheat, maize, and rice are grown as staple foods around the world. Wheat protein content takes an important role in baking and noodle making. High-protein wheat has higher water-absorbing capacity, greater loaf volume, and higher quality potential [11]. Friabilin or puloindolin consist of protein complex around the surface of starch granules. Soft wheats are rich in it; on the contrary, hard wheats contains little, and durum wheat does not contain it [12,13].

The USDA and Health Canada reported that the total dietary fiber contents of wheat ranges from 11 to 12.7% and is composed of both soluble and insoluble fibers. Silano et al. [14] showed that the 0.19 family protein albumin in wheat kernels was a strong inhibitor of α-amylases from human saliva. Moreover, Morimoto et al. [15] showed that wheat albumin inhibits amylases from the human pancreas and saliva, which led to the delay of starch digestion and the suppression of the postprandial abrupt increase in the blood glucose level.

The phenolics are located in the bran layer of the grain kernels, and their functions are caused mainly by ferulic acid, antioxidants cross-linked with arabinoxylans, and produce soluble dietary fiber [16].

Kim and Kweon reported that the quality and noodle-making performance of wheat flour with varied gluten strength is altered by the addition of various arabinoxylans [17].

As the intake of microelements, such as calcium and vitamins, from the daily diet is not sufficient in many countries, many programs for the fortification of calcium [18,19], iron [20], or minerals and vitamins [21] were reported. Particularly, fortification through water seems to be promising because it is an easy, effective, and low-cost method. The relationship between magnesium contents in drinking water and hypomagnesemia was reported [22]. Changes in the mineral composition of food by cooking in hard and soft water was investigated [23]. The relationships between the quality of wheat products, bulgul, acidity, hardness, and iron contents in cooking water was reported [20]. As deep ocean water is rich in minerals, its use was proposed for the fermentation industry and development of functional foods [24]. Utilization of deep ocean water for fermentation of Taiwanese Rice Shochu was investigated [25]. Recently, the prediction method using NIR to predict the mineral composition of wheat flours was developed [26].

Phytic acid has six phosphate groups with a double charge and builds highly insoluble compounds, especially with bivalent metal cations [16]. Zhao et al. [27] reported that phytic acid can effectively improve the appearance of yellow alkaline noodles, which can reduce the formation of browning products. The effects of phosphate salt on the pasting, mixing, and noodle-making performance of wheat flour were reported [28].

Pittas et al. [29] and Yamada and Aoe [30] showed that a combined daily intake of Vitamin D and calcium leads to a potential benefit to reduce the risk of type 2 diabetes.

We reported, in our previous paper, that hard water, rich in Ca, was useful for improving the quality of high-temperature-damaged rice grains, because it inhibited various enzymes (α- and *β*- amylase, proteinase, xylanase), which were activated excessively due to high-temperature damage. Furthermore, calcium intake through the meal was remarkably improved with the boiled rice soaked and cooked using hard water [31,32].

Varietal characteristics affect the quality and processing suitability of wheat flours. For example, usage of primitive wheat flour and whole egg in noodle production was reported [33]. Furthermore, various mutant wheat lines, of which the starch synthase composition differs, showed different starch physicochemical properties and diversified qualities of Chinese noodles [34]. Inokura et al. developed near-isogenic wheat lines, of which starch synthases are diversified, and reported that “Slow Staling” wheat lines from soft wheat are suitable for white salted noodles because they need a short time for cooking, and the texture of the noodle is soft and resistant to hardening after cooking [35]. Li et al. showed that high-amylose wheat has a high amount of resistant starch, and its noodle is slowly digested due to the hard texture [36].

In Japan, most of the wheat cultivars are used as materials for white salted noodle (Udon) and confectionery making. There are not so many wheat varieties with high protein content suitable for bread making. For that reason, it is an important task to improve the taste, functionality, and processing suitability of domestic wheat varieties [37,38]. ‘Sanukinoyume’ is a representative variety for Japanese wheat noodles, and ‘Kitanokaori’ is a newly bred Japanese/Hungarian hybrid variety.

In this work, we evaluated the bio-functionality and eating quality of noodles made from various Japanese wheat flours using weakly acidic hard water.

## 2. Materials and Methods

### 2.1. Materials

Various Japanese wheat flour samples, harvested in 2023, were purchased in 2024 at a local market: Yumechikara (Sapporo, Hokkaido, hard flour), Minaminokaori (Kumamoto, Kumamoto pref., hard flour), Haruyokoi (Sapporo, Hokkaido, hard flour), Kitanokaori (Sapporo, Hokkaido, hard flour), Kitahonami (Sapporo, Hokkaido, medium flour), and Sanukinoyume (Sakaide, Kagawa pref., medium flour) (*n* = 6).

Contrex (hardness: 1468 mg/L; hard water based on WHO standard) (pH 7.4) for cooking noodles and pasting properties with hard water was purchased at a local market in Niigata city. We used purified water (hardness: 17 mg/L; soft water based on WHO standard) (pH 7.6) as a control. In this study, weakly acidic hard water (Contrex pH4.6) was prepared by adjusting the pH with acetic acid.

### 2.2. Measurement of the Moisture Contents of Six Kinds of Wheat Flour

The moisture contents of the wheat sample flours were measured by an oven-drying method by drying 2 g flour samples for 1 h at 135 °C.

### 2.3. Analysis of Phosphorus Contents of Six Kinds of Wheat Flour

The phosphorus contents of 6 kinds of wheat flours were analyzed by the molybdenum blue method [39]. The absorbance was measured at 823 nm, and these measurements were carried out by General Incorporated Association Ken-ou Research Laboratories (Tsubame, Japan).

### 2.4. Preparation of Wheat Starch

Starch granules were prepared from the 6 various flours using the alkaline extraction method under low temperature reported by Yamamoto et al. [40].

### 2.5. Iodine Absorption Spectrum

The AACs (apparent amylose contents) of alkali-treated flours were measured using the iodine spectrophotometric method reported by Juliano [41]. The absorbance was measured at 620 nm (following Juliano’s method), λ_max_ (peak wavelength on iodine staining of starch, which shows a high correlation with the length of the glucan chain; molecular size of amylose and super-long chain (SLC)), and absorbance at λ_max_ (A_λmax_) [42].

A degree of polymerization higher than 37% (Fb_3_) was estimated using the following Equation (1) [42].

Fb_3_ (DP ≧ 37) % = 44.691 × A_λmax_ − 0.774 (1)

### 2.6. Pasting Properties

The pasting properties of 6 kinds of wheat flour samples were measured using a Rapid Visco Analyzer (RVA) (model Super 4 and novel high-pressure-type RVA 4800; Newport Scientific Pty Ltd., Warriewood, Australia). We measured the pasting properties of flour samples using the conditions reported by Toyoshima et al.: the wheat sample (3.5 g: dry weight) was added to 25 mL of purified water or weakly acidic hard water in an aluminum cup, after which the sample cup was installed with the rotor of the RVA, and the wheat flour suspension was heated from 50 °C to 93 °C for 4min after standing for 1 min at 50 °C. The final temperature was held at 93 °C for 7.0 min, cooled from 93 °C to 50 °C for 4 min, and allowed to stand at 50° C for 3 min [43]. The mineral contents of the purified water and hard water are shown in Table S1.

Novel indices such as the ratio of setback to consistency (Set/Cons) were reported to be correlated with Fb_1+2+3_ (DP ≥ 13) [44].

### 2.7. Preparation of Wheat Noodles

Based on the preparation method for rice noodles [32], 6 kinds of wheat flour samples (150 g each) were added to 90 g of purified water, or weakly acidic hard water (Contrex pH 4.6) at 90 °C, followed by kneading for 20 min with hands. Thereafter, the noodles were prepared and boiled as reported in our previous report [32]. These noodle flour samples were pulverized after freeze-drying.

### 2.8. Measurement of Physical Properties of Various Boiled Noodles Using Weakly Acidic Hard Water or Purified Water

The physical properties of wheat noodles were measured by the continuous progressive compression method (CPC) using a Tensipresser (My Boy System, Taketomo Electric Co., Tokyo, Japan), as reported in our previous papers as shown in Figure S1 [45].

### 2.9. Measurement of Color Difference of Various Boiled Flour Noodles Using Weakly Acidic Hard Water or Purified Water

The color differences of boiled wheat needles after using weakly acidic hard water (Contrex; pH 4.6) or purified water were measured using a color difference meter (Color Meter NW-11, Nippon Denshoku Co., Tokyo, Japan).

### 2.10. Analysis of Calcium Contents, RS (Resistant Starch), and FD (Dietary Fiber) of Various Boiled Flour Noodles

The calcium contents of the boiled noodles using weakly acidic hard water (Contrex; pH 4.6) or purified water were analyzed by the dry ashing method and inductively coupled plasma atomic emission spectrometry [32], and those of dietary fiber were measured by microbiological assays and high-performance liquid chromatography–mass spectrometry. These measurements were carried out by General Incorporated Association Ken-ou Research Laboratories. The resistant starch (RS) was measured according to the AOAC method [46] using an RS assay kit (Megazyme, Ltd., Wicklow, Ireland), except the enzyme reaction time. In this study, freeze-dried wheat noodles samples were treated with enzymes (pancreatin and amyloglucosidase) at 36 °C for 6 h [47].

### 2.11. Noodle Making for Feed

The noodle from Kitanokaori using weakly acidic hard water (Contrex; pH 4.6) was used as the test meal (KIT), and the control meal (SAN) was the noodle by Sanukinoyume using purified water. These noodle flour samples were prepared by pulverizing after lyophilization.

### 2.12. Animal Feed Test and Diets

Seven-week-old ICR mice were obtained from Japan SLC Co. Ltd (Hamamatsu, Japan). The mice were housed individually in an air-conditioned room at 20–26 °C under a 12 h light cycle. After acclimatization with a commercial rodent diet (CRF-1, Oriental Yeast, Tokyo, Japan) for 4 days, the mice were divided into two groups of six mice (male) each (weight—control group: 28.5 ± 0.9 g, test group: 28.2 ± 0.9 g) (Test meal: KIT using weakly acidic hard water 50% and starch solution 50 %; Control meal: SAN using purified water 50% and starch solution 50 %; Control meal: SAN 50% and starch solution 50 %). After 20 h fasting, each food was administered at 20 mL/kg liquid volume to mice by single oral administration using a gastric tube. The BGL (blood glucose level) was measured at 0, 30, 60, 90, and 120 min after feeding, using an ACCU-CHEK AVIVA (Roche DC Co., Ltd., Tokyo, Japan) (tail vein blood collection method). The animal feeding test was conducted with the formal approval on Animal Care according to the “Guide for the Care and Use of Laboratory Animals” of the Animal Experimentation Committee, Chitose Research Institute. Measurements of inhibition of the abrupt increase in postprandial blood glucose levels in mice were carried out by the Japan Food Research Laboratories (Chitose, Japan).

### 2.13. Statistical Analyses

For statistical analyses, Excel Statics (ver. 2006; Microsoft Corp., Tokyo, Japan) and Excel NAG Statistics add in 2.0 (The Numerical Algorithms Group Ltd., Tokyo, Japan) were used. For the significance of regression, Student’s *t*-test and Tukey’s multivariate analysis were used.

## 3. Results and Discussion

### 3.1. Moisture and Phosphorus Contents of Six Kinds of Wheat Flour

The moisture content of wheat is closely related to mold growth; therefore, it is desirable for it to be 13.5% or less [48]. The moisture contents of Yumechikara (14.2% ± 0.0), Minaminokaori (14.0% ± 0.0), Haruyokoi (13.8% ± 0.0), and Kitahonami (13.6% ± 0.0) were slightly higher values, whereas those of Sanukinoyume (13.2% ± 0.0) and Kitanokaori (12.9% ± 0.0) were intermediate.

Wheat products provide us with many minerals and trace elements, and their variation is caused by the variety, wheat class, and cultivar, as well as the growing year and location [49]. Balint et al. [50] and Akman and Kara [51] reported that the diploids had higher mineral and trace elements contents than the hexaploids. Minerals and trace elements of wheat are mainly localized in the outer layer of the kernel. American hard wheat has clearly higher contents of most minerals and trace elements than soft-wheat cultivars, and those of durum wheat are lower [49], whereas ‘spelt’ was reported to have clearly higher mineral and trace element contents than many other wheat classes [49]. Blennow et al. [52] showed that wheat starch contains a low level of phosphates, covalently attached to the C-3 and C-6 positions of glucose, largely covalently attached to the amylopectin fraction. Most starches of cereals, roots, tubers, and legumes contain 0.02–0.06% of phosphorus in the form of phospholipid [53].

As shown in Table 1, the phosphorus contents of four Japanese hard-wheat flours in 2023 were 123.5 ± 10.0 mg/100 g (*n* = 4), and those of Japanese medium-wheat flour (*n* = 2) were 83.5 ± 12.0 mg/100g (*n* = 2). In this paper, the phosphorus contents of Japanese hard-wheat cultivars showed a tendency to be higher than the ones in medium-wheat cultivars. Particularly, Sanukinoyume showed the lowest value. As shown in Table S2, calcium, magnesium, iron, and zinc contents indicated a similar tendency. Moreover, the protein contents of Yumechikara (13.5% ± 0.0), Minaminokaori (10.3% ± 0.0), Haruyokoi (11.8% ± 0.0), and Kitanokaori (12.5% ± 0.0) were slightly higher values, whereas those of Sanukinoyume and Kitahonami (9.5% ± 0.0) were intermediate (Table S2) [54].

### 3.2. Iodine Absorption Spectrum for the Survey of Wheat Starch Microstructure

Amylose is one component of starch that greatly affects the quality and gelatinization properties of wheat [55]. Amylose in starches was reported to range from 23.4% to 27.6%, and that of durum wheat showed a slightly higher tendency [56]; it was considered that these cultivars were often influenced by numerous stresses from the environment. Global warming damages grain filling in rice and increases the chalky grains, which deteriorates their cooking and eating qualities, and their AAC showed lower values [31]. AAC consists of a large amount of amylose and a small amount of SLC of amylopectin. Ae mutant rices and high-amylose rice cultivars are too hard and non-sticky for table rice, and their RS contents and pasting temperature are higher than other ordinary cultivars. For this reason, they are promising as low glycemic index (GI) foods.

Components of amylose and amylopectin in principal wheats depend on the genetic origin, which is almost unaffected by environmental conditions [55]. Inouchi et al. [57] and Hirano et al. [58] showed a high positive correlation between the amount of waxy (Wx) protein and SLC contents of starch. Common wheat (*Triticum aestivum* L.) has three Wx proteins, and wheats lacking one or two of the three proteins have been found to show a lower tendency in amylose contents [59]. Takeda et al. [60] showed that cereal amylopectin had a larger number of chains in a cluster than those of root and tuber. Duffus et al. [61] showed that the amylose content of endosperm starch increases during grain development in wheat. Regira et al. [62] developed high-amylose wheat by RNA interference relating branching enzyme (SBEIIa, SBEIIb), and those wheat grains were fed to rats in a diet. As a result, this high-amylose wheat had positive effects on indices of gastrointestinal health in the rats.

As shown in Table 2, the AAC of Minaminokaori had slightly higher values; and those of Haruyokoi, Kitahonami, and Sanukinoyume were next highest; whereas those of Kitanokaori and Yumechikara were intermediate. As a result, there were almost no significant varietal differences in amylose contents.

The difference of λ_max_ values tends to reflect amylose molecular sizes (the length of the glucan chain; molecular size of amylose and SLC of amylopectin) [57]. There were almost no significant varietal difference in λ_max_ values. The λ_max_ value showed a negative correlation with phosphorus contents (r = −0.60; *p* < 0.05).

The Aλ_max_ value reflects not only the properties of amylose but also those of the amylopectin chain length [63]. The Aλ_max_ values of Minaminokaori and Haruyokoi were slightly higher, those of Kitahonami and Sanukinoyume were next highest, and those of Kitanokaori and Yumechikara showed intermediate values. The Aλ_max_ value showed a positive correlation with AAC (r = 0.99; *p* < 0.01).

In our previous study, we showed that the λ_max_/Aλ_max_ ratios in iodine colorimetric measurements were negatively correlated with the apparent amylose content (AAC) [64], and those ratios of low-amylose rice and glutinous rice starches were higher. Therefore, rice cultivars that showed high ratios of λmax/Aλmax were estimated to be palatable and high quality. The λ_max_/Aλ_max_ ratios of Kitanokaori and Yumechikara were very high, and those of Kitahonami and Sanukinoyume were high, whereas Haruyokoi showed an intermediate value, and Minaminokaori showed the lowest value. The λ_max_/Aλ_max_ ratios showed a negative correlation with AAC (r = −0.97; *p* < 0.05).

In our previous study, we developed the novel estimation formulae for the ratio of amylopectin chain lengths, Fb_3_ (degree of polymerization, DP ≥ 37(%)), on the basis of the iodine absorption curve [42]. The Fb_3_ values of Minaminokaori and Haruyokoi were slightly higher, those of Kitahonami and Sanukinoyume were next highest, and those of Kitanokaori and Yumechikara were intermediate. The Fb_3_ (DP ≥ 37)(%), ratio of the proportion of longer amylopectin chains, showed a positive correlation with AAC (r = 0.99; *p* < 0.01).

As a result, Kitanokaori showed a very high ratio of λ_max_/Aλ_max_, which showed to be low-amylose wheat. On the other hand, Sanukinoyume showed a slightly higher AAC, Aλ_max_ value, and Fb_3_ ratio of long glucan chains in amylopectin.

### 3.3. Pasting Properties of Six Kinds of Wheat Flours in Purified Water or in Weakly Acidic Hard Water with an RVA

Physicochemical properties of the starches were often evaluated as pasting characteristics using an RVA, of which analysis was very useful to characterize the starch digestion properties [44,65]. Many investigations have shown that the rheological properties of starch, such as gelatinization, retrogradation, and pasting properties, are affected by amylopectin molecular structure and various amylase activities.

The Final viscosity (Fin. vis) is closely related to the degree of starch retrogradation after cooling. A highly positive relationship was observed between SLC content and consistency (Cons) (= Fin. vis − Mini. vis) [44].

In our previous papers, we reported that it is possible to estimate the amylose content, proportion of intermediate- and long-chains of amylopectin, resistant starch content, and fatty acid [66] composition based upon the pasting properties measured by an RVA.

Takeda et al. [67] reported that wheat starch granules contain about 1% lipid, and the phosphorus is in the form of lysolecithin, some of which is complexed with amylose or outer chains of amylopectin as helical complexes [68,69,70]. Some of the phosphate esters on adjacent amylopectin chains are naturally found cross-linked with various cations, such as calcium and magnesium [71,72]. Substitution of cations from hydrogen ions, etc. to calcium bound to the phosphate was carried out for the purpose of changing the physical properties of starch [73,74].

As the phosphorus contents showed positive correlations with amylose and long chains of amylopectin, we estimate that the effects are mainly due to binding of calcium in hard water and of phosphorus in starch.

As shown in Figure S2, we measured the pasting properties of wheat flour using the same wheat samples for investigating the relationship between phosphorus contents of the starch and calcium included in weakly acidic hard water. Therefore, we used weakly acidic Contrex, pH 4.6 (hardness: 1468 mg/L), or the purified water for the pasting property test and compared the results.

As shown in Table 3 and Table 4, the Max. vis (maximum viscosity) of Sanukinoyume was the highest, and that of Minaminokaori was the lowest value. The Max. vis of wheat flour using weakly acidic hard water was 1.0~1.2 times higher than that in purified water; especially, Minaminokaori showed the highest values. The Max. vis showed a positive correlation with the Fin. vis (final viscosity) (r = 0.90; *p* < 0.01) and Cons (consistency) (r = 0.92; *p* < 0.01), indicators of retrogradation.

The Min. vis (minimum viscosity) of wheat flour using weakly acidic hard water showed lower values than those in purified water: for example, Haruyokoi showed 0.9 times, Kitanokaori 0.9 times, and Sanukinoyume showed 0.5 times. However, that of Minaminokaori was 1.2 times higher compared with the values in purified water. The Min. vis showed significant positive correlations with the Fin. vis (r = 0.98; *p* < 0.01), Cons (r = 0.98; *p* < 0.01), and Max. vis (r = 0.85; *p* < 0.01).

The BD (breakdown; Max. vis–Min. vis) indicates the easiness with which the starch granules are disintegrated [31,75], and that of Sanukinoyume was the highest, and that of Minaminokaori was the lowest. The BD of wheat flour using weakly acidic hard water was 1.0~1.3 times higher than those in purified water; especially, Kitanokaori and Kitahonami showed the highest values. The BD showed a significant positive correlation with the Fin. vis (r = 0.81; *p* < 0.01) and Cons (r = 0.87; *p* < 0.01).

The Fin. vis (final viscosity), indicator of retrogradation, of Sanukinoyume was the highest; that of Kitahonami was the next highest; and that of Minaminokaori showed the lowest value. The Fin. vis of wheat flour using weakly acidic hard water showed lower values than those in purified water: for example, Haruyokoi showed 0.9 times and Sanukinoyume 0.9 times; however, that of Minaminokaori was 1.2 times higher; and those of Yumechikara, Kitanokaorimi, and Kitahonami were almost the same value. The Fin. vis showed a significant positive correlation with the Cons (r = 0.96; *p* < 0.01) and a significant negative correlation with SB (setback) (r = −0.74; *p* < 0.01) and P (phosphorus content) (r = −0.60; *p* < 0.05). Generally, high-amylose cereal starches tend to retrograde more rapidly after gelatinization than the ordinary rice and low-amylose rice [32].

The different peak viscosities (Fin. vis–Max. vis) are shown as “SB” (setback) in this paper, according to the measurements using an RVA. The SB of Sanukinoyume was the lowest, and that of Kitanokaori was the highest. The SB of wheat flour using weakly acidic hard water was 1.3~3.2 times higher than those in purified water; especially, Kitanokaori showed 3.2 times higher values. The SB showed a significant negative correlation with Max. vis (r = −0.96; *p* < 0.01), BD (r = −0.99; *p* < 0.01), Cons (r = −0.80; *p* < 0.01), Fin. vis (r = −0.74; *p* < 0.01), and Min. vis (r = −0.66; *p* < 0.05).

The chain length distribution of amylopectin molecules determines the gelatinization temperature of starch, enthalpy changes, and pasting properties, and the gelatinization temperature of starch increases with the increase in chain length [76].

The Pt of wheat flour using weakly hard water was 1.0~1.2 times higher than those in purified water. The Pt showed a significant positive correlation with the Ca (calcium content) (r = 0.76; *p* < 0.01). As a result, we found that the calcium content of wheat flour showed positive correlations with amylose and long chains of amylopectin in weakly acidic hard water.

The Cons (consistency; Fin. vis–Min. vis), an indicator of retrogradation, of wheat flour using weakly acidic hard water or purified water showed almost the same values. The Cons showed a negative correlation with P (r = −0.59; *p* < 0.05).

In our previous paper, we reported that the novel index of the ratios of SB/Cons, Max/Fin, and Max/Min had higher correlations with RS content, because Fb_1+2+3_ (DP ≧ 13) had a significant positive correlation with SB/Cons and a negative correlation with Max/Fin and Max/Min. The SB/Cons ratios of Kitanokaori showed the highest values, and that of Sanukinoyume was the lowest value. The SB/Cons ratios of wheat flour using weakly acidic hard water was 1.3~3.2 times higher than those in purified water; especially, Kitanokaori showed the highest value. The SB/Cons ratios showed a significant negative correlation with Max/Min (r = −0.94; *p* < 0.01) and Max/Fin (r = −1.00; *p* < 0.01).

In our previous report, we revealed that the phosphorus contents of rice samples correlated significantly with sunlight hours during the seed ripening. We estimated that a high ripening temperature influenced the regulation of genes for starch synthases and branching enzymeⅡb, which lead to a decrease in the amylose content, and in contrast, an increase in long-chain-enriched amylopectin [31].

In the present study, all noodle samples in weakly acidic hard water showed a slightly higher Pt value than those in the purified water due to the binding of calcium in hard water and of phosphorus in starch. Therefore, it seemed that the Pt showed a positive correlation with the calcium content. Moreover, we showed that the phosphorus content of wheat flour samples revealed a significant negative correlation with Fin. vis and Cons., which means that wheat noodles using weakly acidic hard water tend to prevent retrogradation. Furthermore, the BD value of Kitanokaori using weakly acidic hard water was higher than that in purified water, which means there was an improvement of pasting properties by using weakly acidic hard water.

### 3.4. Calcium Contents in Six Kinds of Wheat Flour Noodles Using Weakly Acidic Hard Water (pH 4.6) or Purified Water

It is well known that calcium is an essential mineral for humans. For the prevention of osteoporosis and osteoporotic fractures, it is important to intake calcium efficiently from staple foods. In our previous paper, the calcium and magnesium contents in boiled rice soaked in Contrex (pH 7.2) were 16.5 times or 1.8 times higher than those of boiled rice soaked in purified water. And the calcium and magnesium contents in rice noodles using polished rice flour in Contrex (pH 7.2 and pH4.6) were 5.6–5.8 times or 1.1–1.2 times higher than those of noodles soaked in purified water, respectively. As a result, the calcium and magnesium contents in processed rice products using hard water showed little difference due to different pHs. As magnesium ions are more water-soluble than calcium ions, the magnesium content of boiled rice soaked and boiled in purified water became lower than that of raw polished rice [77,78]. There was an antagonistic effect between calcium and magnesium.

Mineral and trace elements of wheat are mostly situated in the outer part of the grain. The difference in mineral or trace element contents between whole grains and white flour is in most cases two- to fourfold (potassium 2.7-fold, calcium 2.3-fold, copper 2.4-fold) [78]. The ability of dietary fiber to bind (especially) divalent cations such as Ca^2+^, Mg^2+^, Zn^2+^, Cu^2+^, and Fe^2+^ is well known [51].

**Figure 1:**
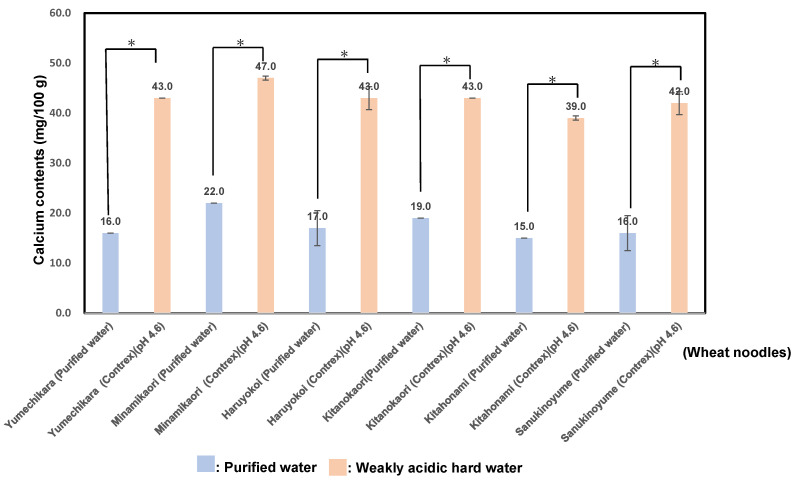
Calcium contents in six kinds of wheat flour noodles using weakly acidic hard water (pH 4.6) or purified water. In results of the comparison between purified water and weakly hard water for producing wheat noodles, for the same wheat sample, (*) denotes statistically significantly differences.

As shown in Figure 1, the calcium contents of six varieties of wheat flour noodles using purified water showed a value of 17.5 ± 2.6 (mg/100 g); particularly, Minaminokaori showed the highest value, 22.0 ± 0.0 (mg/100 g), and those of wheat noodles using wheat flour noodles using weakly acidic hard water showed a value of 42.8 ± 2.6 (mg/100 g); especially, Minaminokaori showed the highest value, 47.0 ± 0.4 (mg/100g). The calcium contents of wheat flour noodles using weakly acidic hard water showed values 2.1~2.7 times higher than those in purified water; especially, Yumechikara showed the highest ratio.

As a result, it seemed that the calcium content of wheat noodles using weakly acidic hard water showed a similar tendency as ones using purified water. Perhaps the characteristics of wheat grains using weakly acidic hard water were caused by the difference in the fine structure of amylopectin with enriched long chains within a cluster. In the present study, hard-wheat cultivars showed a slightly higher calcium content than those of medium ones. The wheat noodles using weakly acidic hard water are useful for increasing calcium intake through the meal. A new noodle that can compensate for calcium deficiency was developed with weakly acidic hard water.

The bioavailability of minerals and trace elements from wheat and wheat products is associated with dietary fiber, which has potent cation-exchanging capacity and may therefore have a negative effect on the bioavailability of minerals and trace elements [16]. It has been found that if the amount of dietary fiber in the diet is 10 to 20%, the absorption of potassium, calcium, magnesium, and phosphorus decrease by about 10% [79]. On the contrary, the calcium content of our noodles prepared using weakly acidic hard water was about three times that of noodles using purified water. It seems that long-term animal tests (for example, bone density changes in mice) and human trials should be performed in the future.

### 3.5. Textural Properties of Six Kinds of Domestic Wheat Flour Noodles Using Weakly Acidic Hard Water (pH 4.6) or Purified Water

Previous studies reported that physical properties of Japanese wheat dough and boiled noodles have a tendency to be weaker compared with Australian standard white (ASW), because Japanese domestic wheat flour showed rather lower amylose and protein content than that of ASW [65,80]. Toyokawa et al. [38] showed that the important quality attributes of wheat flour noodles are color, taste, surface appearance upon cooking, and eating quality, deriving principally from the characteristics of starch [81]. Crosbie et al. [82] showed that the swelling volume of Japanese wheat flour shows a positive correlation with the total texture score and its attributes, namely, the balance of softness to hardness, elasticity, and smoothness [82,83].

We reported that the boiled rice grains boiled in hard water showed slightly higher hardness, toughness, stickiness, and cohesiveness compared with ones boiled in purified water, because various hydrolytic enzymes were inhibited by boiling in hard water [31].

And weakly acidic hard water (pH 4.6) showed higher α-amylase activity levels than those of hard water (pH 7.2); however, the hardness of boiled rice with high amylose and *ae* mutant rice boiled in hard water (pH 4.6) showed an almost similar tendency to that in f hard water (pH 7.2) [78].

We measured and compared the textural properties of six kinds of wheat noodles using weakly acidic hard water (pH 4.6) or using purified water. Measurements of the physical properties of the wheat flour noodles by CPC with the Tensipresser is shown in Figure S2.

Noda et al. [84,85] reported that calcium-fortified potato starch showed strong ionic binding with starch phosphate; therefore, the characteristics showed a similar tendency to the modified starch of phosphate cross-linked starch.

As shown in Table 5, for the Tenderness (softness) of wheat noodles using purified water, the highest was Sanukinoyume, followed in order by Kitahonami, Yumechikara, Kitanokaori, Minaminokaori, and Haruyokoi, and for the Toughness (strength), the highest was Sanukinoyume, followed in order by Yumechikara, Minaminokaori, Kitanokaori, Kitahonami, and Haruyokoi. The Tenderness and Toughness of wheat noodles prepared using weakly acidic hard water was a little lower than those using purified water. As shown in Table 4, the Tenderness showed a positive correlation with Toughness (r = 0.91; *p* < 0.01), Min. vis (r = 0.66; *p* < 0.05), and Fin. vis (r = 0.69; *p* < 0.05) and a negative correlation with P (phosphorus contents) (r = −0.78; *p* < 0.01).

The Hardness of wheat noodles using weakly acidic hard water or purified water showed little difference. The Hardness showed a negative correlation with Max. vis (r = −0.63; *p* < 0.05), Min. vis (r = −0.66; *p* < 0.05), Fin. vis (r = −0.72; *p* < 0.01), and Cons (r = −0.75; *p* < 0.01).

The Pliability (flexibility) and Brittleness of wheat noodles using weakly acidic hard water showed almost the same values as ones using purified water. The Pliability showed a negative correlation with RS (resistant starch) (r = −0.78; *p* < 0.01), and the Brittleness showed a negative correlation with Hardness (r = −0.69; *p* < 0.05).

In this study, the physical properties of wheat flour noodles using purified water or weakly acidic hard water showed no significant differences.

### 3.6. Improvement of the Color of Six Kinds of Wheat Noodles Using Weakly Acidic Hard Water, Contrex (pH 4.6)

In our previous paper, the yellowish degree (ratio of color difference b*) of boiled rice soaked in weakly acidic hard water (pH 4.6) showed 0.4 times lower b* values than those of purified water [77]. Color is an important quality criterion for Japanese noodles [86]. Lutein, one of the carotenoids, contributes principally to the color of noodles (whiteness, brightness, yellowness) [87]. Hou et al. [88] and Ito et al. [89] reported that color and physical properties change depending on the difference in the polyphenol-oxidase, yellow pigment, ash and protein contents, and color of flours.

As shown in Table 6, we evaluated the color difference of domestic wheat flour noodles using weakly acidic hard water (pH 4.6) or purified water. The WB (whiteness) of wheat flour noodles using weakly acidic hard water was 1.04~1.25 times higher than those in purified water; especially, Yumechikara showed the highest values, and L*(brightness) showed a similar tendency.

Moreover, a ratio of the color difference (ΔE^*^(ab)) of wheat noodles using weakly acidic hard water was 0.88~0.98 times lower than those in purified water, and those of b* showed a similar tendency.

Also, the reddish degree (ratio of color difference a*) of wheat noodles using weakly acidic hard water was slightly lower compared to the wheat noodles using purified water.

### 3.7. Measurement of Bio-Functional Properties of RS (Resistant Starch) and Dietary Fiber of Six Kinds of Wheat Flour Noodles Using Weakly Acidic Hard Water or Purified Water

Sajilate et al. [90] showed that it is possible to classify RS (resistant starch) into four types. Type 4 is chemically modified starch, which interferes with enzymatic digestion [91]. The distarch phosphate potato starch, and that of rice, tapioca starch, sweet potato starch, and wheat starch, are sources for RS Type 4 [92].

In our previous paper, we reported that the textural qualities of boiled rice using weakly acid Contrex (pH 4.6 and pH 7.2) showed an almost similar tendency [77].

**Figure 2:**
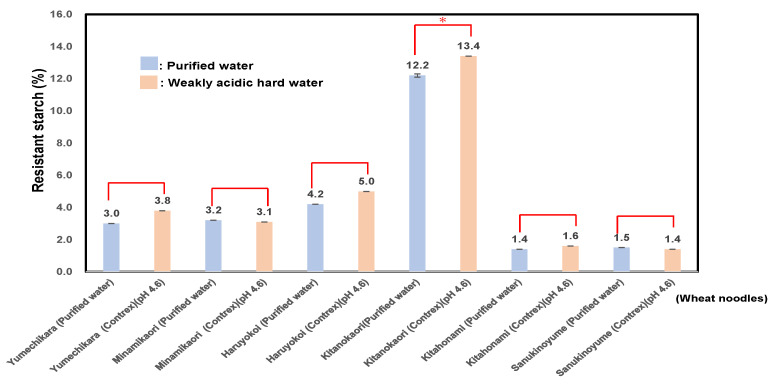
RS contents of six kinds of wheat noodles using weakly acidic hard water (pH 4.6) or purified water. In results of the comparison between purified water and weakly hard water for producing wheat noodles, for the same wheat sample, (*) denotes statistically significant differences.

As shown in Figure 2, the RS contents of six varieties of wheat noodles using purified water showed a value of 4.3 ± 4.0 (%): particularly, Kitanokaori showed the highest value 12.2 ± 0.1 (%), and those of wheat noodles using weakly acidic hard water showed a value of 4.7 ± 4.4 (%); especially, Kitanokaori showed the highest values 13.4 ± 0.0 (%). The RS contents of wheat noodles using weakly acidic hard water were 0.9~1.3 times higher than those prepared in purified water. As a result, it rather seemed that the calcium-fortified wheat starch showed a little ionic binding with wheat starch phosphate in weakly acidic hard water, and those characteristics showed a similar tendency to the modified starch of phosphate cross-linked starch.

As shown in Table 4, RS contents showed a significant positive correlation with the P (r = 0.58; *p* < 0.05). It seemed that the phosphorus contents of wheat flour samples revealed a significant negative correlation with Fin. vis and Cons., an indicator of retrogradation.

It was shown that the calcium contents in the noodles effectively increased by using weakly acidic hard water, and also the RS contents of wheat noodles using weakly acidic hard water tended to be higher than those prepared in purified water.

Tabiki et al. [93] reported that the pedigree of “Kitanokaori” is “Horoshiri komugi”/“GK Szemes”. GK Szemes is a Hungarian wheat variety, which has good bread-making quality, and Kitanokaori showed high milling quality, bread-making quality, and high pentosan contents, which exceed those of the parents.

As a result, the RS contents of noodles from Kitanokaori using weakly acidic hard water were 2.9~8.4 times higher than those other cultivars using weakly acidic hard water.

Pentosans are the major non-starch polysaccharides of wheat flours [94]. Wheat flour contains water-soluble and water-insoluble pentosans. The soluble pentosans are composed of arabinoxylans, which produce some short-chain fatty acids by the gut microbial fermentation. Therefore, they are one of the main components of dietary fiber in cereals [95,96]. Shogren et al. [97] showed that water-soluble and water-insoluble pentosans improve bread-making properties of wheat flours. It seemed that various wheat cultivars have different cell-wall properties based on the cell-wall components, sugar linkages, etc. [98].

**Figure 3:**
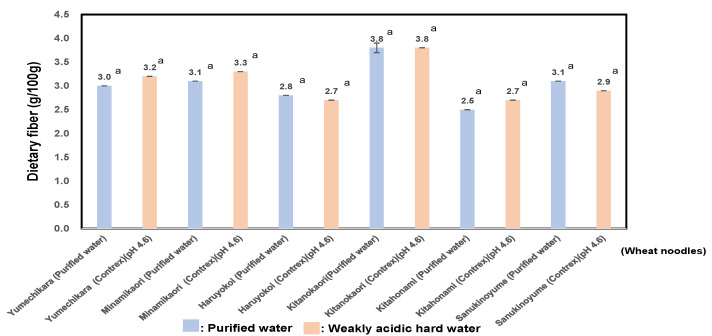
Dietary fiber of six kinds of wheat noodles using weakly acidic hard water (pH 4.6) or purified water. In results of the comparison between purified water and weakly hard water for producing wheat noodles, for the same wheat sample, letter (a) denotes statistically significant differences.

As shown in Figure 3, the dietary fiber of six varieties of wheat noodles using purified water showed values of 2.5 ± 3.8 (g/100 g); particularly, Kitanokaori showed the highest value, 3.8 ± 0.1 (g/100 g), and those of wheat noodles using weakly acidic hard water showed values of 2.7 ± 3.3 (g/100 g); especially, Kitanokaori showed the highest values, 3.8 ± 0.0 (g/100 g). The dietary fiber of wheat noodles using weakly acidic hard water showed almost the same values as ones using purified water. As shown in Table 4, the dietary fiber showed a significant positive correlation with the RS (r = 0.82; *p* < 0.01).

Among the six wheat cultivars, Kitanokaori was shown to be a characteristic wheat cultivar in terms of bio-functionality, because it contains the most amount of resistant starch and dietary fiber.

### 3.8. Determination of the Initial BGL of Aged Mice Kept for 8 Weeks

Yamanaka and Aoe reported that in KK Mice fed a diet low in calcium, their pancreas induced inflammation, and their insulin secretion showed a lower tendency [29]. Ogata et al. [99] and Farlay et al. [100] reported that osteoporosis is one of the complications of diabetes. Moreover, Oei et al. [101] showed that poor glycemic control in type 2 diabetes is associated with fracture risk. Villegas et al. [9] and Liu et al. [10] reported that it is important to consume adequate amounts of calcium for diabetes prevention.

RS and dietary fiber are the same undigestible polysaccharide, which are derived from starch or non-starch polysaccharide, and which have similar nutritional physiology, for example, inhibition for blood sugar elevation and cholesterol-lowering effect.

**Figure 4:**
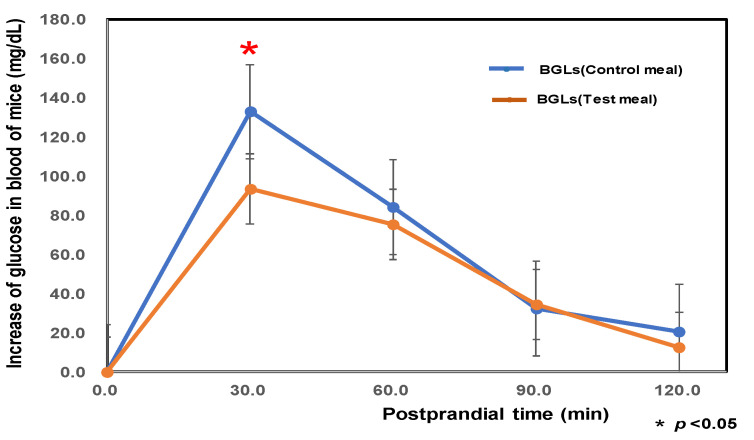
The initial blood glucose level after the fasting period of 20 h of aged mice after 8 weeks. Control meal: SAN 50% and starch solution 50%; SAN noodle from Sanukinoyume (Japanese premium wheat flour) using purified water. Test meal: KIT 50% and starch solution 50%; KIT noodle from Kitanokaori (newly developed Japanese wheat flour) using weakly acid hard water. Mice group size (*n* = 6). (*) denotes statistically significantly differences.

As shown in Figure 4, we prepared noodle (KIT) from Kitanokaori (newly developed Japanese wheat flour) using weakly acidic hard water, which showed greater resistant starch (9.0-fold), dietary fiber (1.2-fold), and calcium (2.7-fold) contents than noodle (SAN) from Sanukinoyume (Japanese premium wheat flour) using purified water. Furthermore, aged mice, which were fed a KIT diet for eight weeks, showed lower postprandial blood glucose levels (BGLs) after consumption at 30 min than mice fed a control diet (SAN); in detail, the increase in glucose in blood for the control sample was 132.7 ± 34.0 (mg/dL), and that of the test sample was 93.2 ± 22.3 (mg/dL) (significant at 95%, 4.52 < *p* = 5.10 < 5.39).

Furthermore, Kitanokaori has superior characteristics, such as resistance to leaf rust and powdery mildew, good bread-making quality, and high lodging resistance compared to other wheat cultivars in Hokkaido [94]. For this reason, we recommended Kitanokaori as a material for the development of palatable and functional wheat noodles.

Although existing wheat products, such as whole wheat flour noodle, have a lot of merits, for example, high amounts of nutritional components, low cost, and easy handling, our KIT noodle using weakly acidic hard water also has a lot of merits, such as a high amount of resistant starch, calcium, bright color, and good texture. It seems necessary to overcome the cost for the preparation of weakly acidic hard water.

## 4. Conclusions

Type 2 diabetes and osteoporosis are very serious diseases all over the world. In this paper, we reported that a newly developed wheat cultivar, Kitanokaori, contains more resistant starch than the other wheat cultivars. In terms of the pasting properties of wheat flour and the textural properties of the noodle from Kitanokaori (KIT), they are not inferior to those of the traditional premium wheat cultivar, Sanukinoyume. Furthermore, we found that KIT using weakly acidic hard water contained a remarkably high amount of resistant starch, dietary fiber, and calcium content. We proved that KIT inhibits a postprandial abrupt increase in blood glucose in mice. Therefore, KIT seems to be promising as a functional food, by which type 2 diabetes and osteoporosis could be prevented.

## Figures and Tables

**Table 1:** Phosphorus contents of six kinds of Japonica wheat flour.

	Phosphorus
	contents
	(mg/100 g)
Yumechikara	132.0 ± 2.6 a
Minaminokaori	109.0 ± 1.5 c
Haruyokoi	126.0 ± 3.2 b
Kitanokaori	127.0 ± 3.0 b
Kitahonami	92.0 ± 2.1 d
Sanukinoyume	75.0 ± 1.8 e

Different letters (a, b, c, d, e) denote statistically significant differences. Values are shown as mean ± standard deviation.

**Table 2:** The analysis of iodine absorption parameters of the starch of six varieties of wheat starches.

Cultivars	AAC	λ_max_	A_λmax_	λ_max/_	Fb_3_
	(%)			A_λmax_	(DP ≧ 37) (%)
Yumechikara	25.6 ± 0.5 c	598.0 ± 2.8 a	0.440 ± 0.003 c	1359.1 ± 2.3 a	18.9 ± 0.1 b
Minamnoikaori	28.0 ± 0.6 a	601.0 ± 2.8 a	0.469 ± 0.004 a	1282.8 ± 3.6 d	20.2 ± 0.2 a
Haruyokoi	27.2 ± 0.1 b	599.0 ± 1.4 a	0.461 ± 0.001 a	1300.8 ± 5.1 c	19.8 ± 0.0 a
Kitanokaori	25.9 ± 0.3 c	600.0 ± 0.0 a	0.441 ± 0.004 c	1360.6 ± 13.1 a	18.9 ± 0.2 b
Kitahonami	26.7 ± 0.3 b	601.5 ± 0.7 a	0.450 ± 0.005 b	1338.2 ± 16.3 b	19.3 ± 0.2 b
Sanukinoyume	26.6 ± 0.7 b	600.0 ± 2.8 a	0.450 ± 0.006 b	1333.4 ± 10.5 b	19.3 ± 0.3 b

Within each value (AAC, λ_max_, Aλ_max_, etc.) in the same column and in each sample, different letters (a, b, c, d) denote statistically significant differences. AAC: apparent amylose content. Values are shown as mean ± standard deviation.

**Table 3:** Pasting properties of six kinds of domestic wheat flour using purified water or weakly acidic hard water with an RVA.

	Max. vis	Mini. vis	BD	Fin. vis	SB
	(cP)	(cP)	(cP)	(cP)	(cP)
Yumechikara (Purified water)	2868.0 ± 25.5 a	1263.5 ± 10.6 a	1604.5 ± 14.8 b	2609.5 ± 7.8 a	−258.5 ± 17.7 a
Yumechikara (Contrex) (pH 4.6)	3165.0 ± 227.7 a	1206.5 ± 46.0 b	1958.5 ± 181.7 a	2528.0 ± 113.1 a	−637.0 ± 114.6 b
Minamikaori (Purified water)	1781.5 ± 36.1 b	844.5 ± 26.2 a	937.0 ± 9.9 a	1991.0 ± 62.2 b	−209.5 ± 26.2 a
Minamikaori (Contrex) (pH 4.6)	2098.0 ± 4.2 a	1173.0 ± 5.7 b	925.0 ± 9.9 a	2369.0 ± 5.7 a	−271.0 ± 1.4 b
Haruyokoi (Purified water)	3139.0 ± 7.1 b	1304.0 ± 17.0 a	1835.0 ± 9.9 b	2662.0 ± 25.5 a	−477.0 ± 18.4 a
Haruyokoi (Contrex) (pH 4.6)	3230.5 ± 2.1 a	1181.5 ± 13.4 b	2049.0 ± 15.6 a	2486.5 ± 16.3 b	−744.0 ± 18.4 b
Kitanokaori (Purified water)	2772.0 ± 38.2 b	1214.5 ± 10.6 a	1557.5 ± 27.6 b	2589.5 ±23.3 a	−182.5 ± 14.8 a
Kitanokaori (Contrex) (pH 4.6)	3091.0 ± 26.9 a	1115.0 ± 7.1 b	1976.0 ± 19.8 a	2513.5 ± 13.4 b	−577.5 ± 13.4 b
Kitahonami (Purified water)	3177.0 ± 19.8 b	1381.5 ± 4.9 a	1795.5 ± 14.8 b	2834.5 ± 6.4 a	−342.5 ± 13.4 a
Kitahonami (Contrex) (pH 4.6)	3642.5 ± 244.0 a	1386.5 ± 47.4 a	2256.0 ± 196.6 a	2856.0 ± 93.3 a	−786.5 ± 150.6 b
Sanukinoyume (Purified water)	4068.0 ± 9.9 b	1580.5 ± 19.1 a	2487.5 ± 9.2 b	3163.0 ± 32.5 a	−905.0 ± 22.6 a
Sanukinoyume (Contrex) (pH 4.6)	4107.0 ± 1.4 a	868.8 ± 0.4 b	2706.0 ± 14.1 a	2905.5 ± 2.1 b	−1201.5 ± 3.5 b
	**Pt**	**Cons**	**Set/Cons**	**Max/Min**	**Max/Fin**
	**(°C)**	**(°C)**			
Yumechikara (Purified water)	50.5 ± 0.3 b	1346.0 ± 2.8 a	−0.19 ± 0.01 a	2.27 ± 0.00 b	1.10 ± 0.01 b
Yumechikara (Contrex) (pH 4.6)	59.7 ± 0.5 a	1321.5 ± 67.2 a	−0.48 ± 0.06 b	2.62 ± 0.09 a	1.25 ± 0.03 a
Minamikaori (Purified water)	53.5 ± 4.7 a	1146.5 ± 36.1 b	−0.18 ± 0.02 a	2.11 ± 0.02 a	0.89 ± 0.01 a
Minamikaori (Contrex)(pH 4.6)	59.5 ± 0.6 a	1196.0 ± 11.3 a	−0.23 ± 0.00 b	1.79 ± 0.01 b	0.89 ± 0.00 a
Haruyokoi (Purified water)	50.7 ± 0.0 b	1358.0 ± 8.5 a	−0.35 ± 0.02 a	2.41 ± 0.03 b	1.18 ± 0.01 b
Haruyokoi (Contrex) (pH 4.6)	59.5 ± 0.3 a	1305.0 ± 2.8 b	−0.57 ± 0.02 b	2.73 ± 0.03 a	1.30 ± 0.01 a
Kitanokaori (Purified water)	55.7 ± 0.1 a	1375.0 ± 12.7 a	−0.13 ± 0.01 a	2.28 ± 0.01 b	1.07 ± 0.01 b
Kitanokaori (Contrex) (pH 4.6)	58.0 ± 1.3 b	1398.5 ± 6.4 a	−0.41 ± 0.01 b	2.77 ± 0.01 a	1.23 ± 0.00 a
Kitahonami (Purified water)	51.2 ± 0.9 b	1453.0 ± 1.4 a	−0.24 ± 0.01 a	2.30 ± 0.01 b	1.12 ± 0.00 b
Kitahonami (Contrex) (pH 4.6)	59.7 ± 0.6 a	1469.5 ± 46.0 a	−0.53 ± 0.09 b	2.63 ± 0.09 a	1.27 ± 0.04 a
Sanukinoyume (Purified water)	50.2 ± 0.0 b	1582.5 ± 13.4 a	−0.57 ± 0.02 a	2.57 ± 0.02 b	1.29 ± 0.01 b
Sanukinoyume (Contrex) (pH 4.6)	59.4 ± 0.6 a	1504.5 ± 10.6 b	−0.80 ± 0.00 b	2.93 ± 0.03 a	1.41 ± 0.00 a

Within each value (Max. vis, Min. vis, BD, etc.) in the same column and between using purified water or weakly acidic hard water in each sample, different letters (a, b) denote statistically significantly differences. RVA, rapid visco analyzer; SB, setback; BD, breakdown; Max. vis., maximum viscosity; Mini. vis., minimum viscosity; Pt, pasting temperature; Cons, consistency; Fin vis., final viscosity. Values are shown as mean ± standard deviation.

**Table 4:** Correlation between the RS contents, phosphorus contents, calcium contents, textural properties of noodles, pasting properties, and iodine absorption curve of six kinds of domestic wheat flour samples.

	DF	RS	P	Ca	Tende	Pliab	Tough	Britt	Hard	Max. vis	Mini. vis	BD	Fin. vis
Dietary fiber	1.00												
RS	0.82 **	1.00											
P	0.38	0.58 *	1.00										
Ca	0.15	0.11	0.06	1.00									
Tenderness	−0.14	−0.38	−0.78 **	−0.35	1.00								
Pliability	−0.51	−0.71 **	−0.39	0.04	0.08	1.00							
Toughness	0.02	−0.32	−0.57	−0.32	0.91 **	0.05	1.00						
Brittleness	−0.30	−0.19	−0.37	0.18	−0.06	0.01	−0.28	1.00					
Hardness	0.18	−0.04	0.44	0.04	−0.24	0.29	0.13	−0.69 *	1.00				
Max. vis	−0.30	−0.23	−0.52	0.04	0.57	−0.25	0.39	0.39	−0.63 *	1.00			
Mini. vis	−0.35	−0.39	−0.57	−0.20	0.66 *	−0.21	0.46	0.37	−0.66 *	0.85 **	1.00		
BD	−0.27	−0.16	−0.46	0.12	0.50	−0.25	0.34	0.37	−0.57	0.98 **	0.74 **	1.00	
Fin. vis	−0.29	−0.29	−0.60 *	−0.20	0.69 *	−0.27	0.48	0.40	−0.72 **	0.90 **	0.98 **	0.81 **	1.00
SB	0.28	0.16	0.41	−0.21	−0.42	0.22	−0.29	−0.34	0.50	−0.96 **	−0.66 *	−0.99 **	−0.74 **
Pt	0.19	−0.17	−0.06	0.76 **	−0.28	0.24	−0.23	0.29	−0.01	0.03	−0.09	0.07	−0.14
Cons	−0.18	−0.13	−0.59 *	−0.19	0.69 *	−0.33	0.47	0.42	−0.75 **	0.92 **	0.89 **	0.87 **	0.96 **
Set/cons	0.28	0.12	0.32	−0.22	−0.34	0.26	−0.22	−0.33	0.48	−0.94 **	−0.63*	−0.98 **	−0.70 *
Max/Min	−0.11	0.09	−0.19	0.30	0.20	−0.29	0.11	0.27	−0.39	0.80 **	0.37	0.89 **	0.49
Max/Fin	−0.26	−0.09	−0.29	0.24	0.32	−0.28	0.20	0.32	−0.49	0.93 **	0.62 *	0.97 **	0.69 *
AAC	−0.31	−0.36	−0.26	0.10	−0.15	0.56	−0.15	−0.06	0.24	−0.45	−0.32	−0.46	−0.40
λ_max_	−0.11	−0.14	−0.60 *	0.01	0.17	0.30	−0.06	0.47	−0.42	−0.13	0.00	−0.17	0.02
A_λmax_	−0.32	−0.36	−0.17	0.10	−0.19	0.56	−0.15	−0.15	0.34	−0.46	−0.35	−0.46	−0.44
λ_max_/A_λmax_	0.33	0.37	0.13	−0.10	0.21	−0.55	0.15	0.19	−0.38	0.45	0.35	0.45	0.44
Fb_3_	−0.32	−0.36	−0.17	0.10	−0.19	0.56	−0.15	−0.15	0.34	−0.46	−0.35	−0.46	−0.44
	**SB**	**Pt**	**Cons**	**Set/Cons**	**Max/Min**	**Max/Fin**	**AAC**	**λ_max_**	**A_λmax_**	**λ_max_/A_λmax_**	**Fb_3_**		
SB	1.00												
Pt	−0.15	1.00											
Cons	−0.80 **	−0.21	1.00										
Set/cons	0.99 **	−0.16	−0.76 **	1.00									
Max/Min	−0.93 **	0.13	0.63 *	−0.94 **	1.00								
Max/Fin	−0.99 **	0.16	0.75 **	−1.00**	0.95 **	1.00							
AAC	0.42	0.17	−0.49	0.46	−0.47	−0.47	1.00						
λ_max_	0.23	0.06	0.06	0.29	−0.28	−0.30	0.53	1.00					
A_λmax_	0.42	0.18	−0.54	0.45	−0.46	−0.46	0.99 **	0.39	1.00				
λ_max_/A_λmax_	−0.40	−0.18	0.55	−0.42	0.44	0.44	−0.97 **	−0.32	−1.00 **	1.00			
Fb_3_	0.42	0.18	−0.54	0.45	−0.46	−0.46	0.99 **	0.39	1.00	−1.00 **	1.00		

Correlation is significant at 5% (*) or 1% (**) by the method of *t*-test. DF: dietary fiber; RS: resistant starch; P: phosphorus; Tende: tenderness; Pliab: pliability; Tough: toughness; Britt: brittleness; Hard: hardness.

**Table 5:** Textural properties of six kinds of domestic wheat flour noodles using weakly acidic hard water (pH 4.6) and purified water.

	Tenderness	Pliability	Toughness	Brittleness	Hardness
	(N/cm^2^)		(N/cm^2^)		(N/cm^2^)
Yumechikara (Purified water)	910.9 ± 142.7 a	1.02 ± 0.11 a	357.2 ± 61.4 a	1.75 ± 0.17 a	17.33 ± 2.52 a
Yumechikara (Contrex) (pH 4.6)	691.6 ± 108.4 b	0.99 ± 0.01 a	268.6 ±49.1 b	1.73 ± 0.07 a	16.67 ± 0.58 a
Minamikaori (Purified water)	762.7 ± 48.4 a	1.12 ± 0.02 a	304.8 ± 2.8 a	1.53 ± 0.03 a	19.33 ± 1.15 a
Minamikaori (Contrex) (pH 4.6)	744.0 ± 63.7 a	1.06 ± 0.06 a	266.3 ± 63.4 a	1.79 ± 0.28 a	16.33 ± 3.51 a
Haruyokoi (Purified water)	611.4 ± 62.9 a	1.00 ± 0.04 a	184.3 ± 18.9 b	2.05 ± 0.06 a	13.33 ± 0.58 a
Haruyokoi (Contrex) (pH 4.6)	675.7 ± 58.1 a	0.97 ± 0.04 a	268.2 ± 37.6 a	1.76 ± 0.16 a	16.67 ± 1.53 a
Kitanokaori (Purified water)	841.1 ± 218.8 a	0.92 ± 0.06 a	302.0 ± 117.9 a	2.09 ± 0.41 a	14.00 ± 3.61 a
>Kitanokaori (Contrex) (pH 4.6)	714.6 ± 91.8 a	0.91 ± 0.02 a	229.7 ± 26.8 a	2.15 ± 0.19 a	13.00 ± 2.00 a
Kitahonami (Purified water)	1106.4 ± 457.0 a	1.04 ± 0.03 a	289.5 ± 149.6 a	2.42 ± 0.67 a	11.33 ± 3.51 a
Kitahonami (Contrex) (pH 4.6)	727.8 ± 447.8 b	1.02 ± 0.06 a	212.9 ± 146.9 b	5.35 ± 4.70 a	10.67 ± 7.51 a
Sanukinoyume (Purified water)	1530.8 ± 329.3 a	0.96 ± 0.03 a	539.1 ± 181.2 a	1.94 ± 0.31 a	14.00 ± 3.00 a
Sanukinoyume (Contrex) (pH 4.6)	1250.5 ± 638.0 a	1.10 ± 0.10 a	392.2 ± 226.1 b	2.37 ± 1.05 a	13.33 ± 6.11 a

Within each value (Tenderness, Pliability, etc.) in the same column and between using purified water or weakly hard water in each sample, different letters (a, b) denote statistically significantly differences. Values are shown as mean ± standard deviation.

**Table 6:** Color differences of six kinds of wheat noodles using weakly acidic hard water (pH 4.6) or purified water.

	WB	ΔE (ab)	a*	b*
Yumechikara (Purified water)	19.6 ± 2.0 b	42.0 ± 2.0 a	−1.1 ± 0.1 a	13.2 ± 0.7 a
Yumechikara (Contrex) (pH 4.6)	24.5 ± 3.2 a	37.2 ± 2.9 b	−1.4 ± 0.3 a	12.8 ± 0.8 b
Minamikaori (Purified water)	17.3 ± 1.1 b	44.3 ± 1.2 a	−1.2 ± 0.0 a	14.5 ± 0.3 a
Minamikaori (Contrex) (pH 4.6)	21.3 ± 0.9 a	40.1 ± 1.0 b	−1.2 ± 0.0 a	13.8 ± 0.2 b
Haruyokoi (Purified water)	21.4 ± 1.2 a	40.4 ± 0.1 a	−1.3 ± 0.0 a	12.1 ± 0.2 a
Haruyokoi (Contrex) (pH 4.6)	22.3 ± 1.0 a	39.6 ± 0.1 a	−1.1 ± 0.0 a	11.6 ± 0.1 b
Kitanokaori(Purified water)	22.2 ± 0.0 a	39.1 ± 0.0 a	−1.3 ± 0.1 a	14.1 ± 0.3 a
Kitanokaori (Contrex) (pH 4.6)	22.3 ± 0.1 a	38.0 ± 0.1 b	−1.4 ± 0.1 a	14.7 ± 0.8 a
Kitahonami (Purified water)	21.5 ± 1.5 b	40.8 ± 2.1 a	−2.1 ± 0.1 a	10.6 ± 1.8 a
Kitahonami (Contrex) (pH 4.6)	26.2 ± 1.0 a	36.1 ± 0.7 b	−2.0 ± 0.1 a	10.8 ± 0.8 a
Sanukinoyume (Purified water)	24.2 ± 1.7 b	34.7 ± 1.4 a	−2.3 ± 0.1 a	13.6 ± 0.3 a
Sanukinoyume (Contrex) (pH 4.6)	29.8 ± 1.6 a	33.0 ± 1.2 b	−2.5 ± 0.0 a	11.3 ± 0.3 b

Within each value (WB, ΔE (ab), a*, b*, etc.) in the same column and between using purified water or weakly hard water in each sample, different letters (a, b) denote statistically significantly differences. Values are shown as mean ± standard deviation.

## Data Availability

The data presented in this study are available on request from the corresponding author due to the ethical statement request for the animal.

## Supplementary Materials

The following supporting information can be downloaded at: https://www.mdpi.com/article/10.3390/foods14061044/s1, Figure S1: The physical properties of wheat noodles were measured by the continuous progressive compression method (CPC). Figure S2: Pasting properties of six kinds of domestic wheat flour using purified water or weakly acidic hard water with an RVA. Continuous progressive compression test (CPC-test). Table S1. The mineral contents of purified water or hard water. Table S2. The principal ingredient of six kinds of Japonica wheat flour.

## Abbreviations

AAC: apparent amylose content; RS, resistant starch; SLC, super-long chain; DP, degree of polymerization; RVA, rapid visco analyzer; SB, setback; BD, breakdown; Max. vis., maximum viscosity; Mini. vis., minimum viscosity; Pt, pasting temperature; Cons, consistency; Fin vis., final viscosity; KIT, noodle from ‘Kitanokaori’; SAN, noodle from ‘Sanukinoyume’; DF: dietary fiber; P: phosphorus.

## References

[1] The IDF (International Diabetes Federation) Diabetes Atlas 10th. https://diabetesatlas.org/date/en/.

[2] (2015). ADI (Alzheimer’s Disease International) Report. https://www.alzint.org/resource/world-alzheimerreport-2015/.

[3] Nakamura S., Ikeuchi T., Araki A., Kasuga K., Watanabe K., Hirayama M., Ito M., Ohtsubo K. (2022). Possibility for prevention of type 2 diabetes mellitus and dementia using three kinds of brown rice blends after high-pressure treatment. Foods.

[4] Tomaru M., Takano H., Osakabe N., Yasuda A., Inoue K., Yanagisawa R., Ohwatari T., Uematsu H. (2007). Dietary supplementation with cacao liquor proanthocyanidins prevents elevation of blood glucose levels in diabetic obese mice. Nutrition.

[5] Kanamoto Y., Yamashita Y., Nanba F., Yoshida T., Tsuda T., Fukuda I., Nakamura-Tsuruta S., Ashida H. (2011). A black soybean seed coat extract prevents obesity and glucose intolerance by up-regulating uncoupling proteins and down-regulating inflammatory cytokines in high-fat diet-fed mice. J. Agric. Food Chem..

[6] Vestergaard P. (2007). Discrepancies in bone mineral density and fracture risk in patients with type 1 and type 2 diabetes—A meta-analysis. Osteoporos. Int..

[7] Parthasarathy S., Khoo J.C., Miller E., Barnett J., Witztum J.L., Steinberg D. (1990). Low density lipoprotein rich in oleic acid is protected against oxidative modification: Implications for dietary prevention of atherosclerosis. Proc. Natl. Acad. Sci. USA.

[8] Nakajima M., Cooney M.J., Tu A.H., Chang K.Y., Cao J., Ando A., An G.J., Melia M., de Juan E. (2001). Normalization of retinal vascular permeability in experimental diabetes with genistein. Investig. Ophthalmol. Vis. Sci..

[9] Villegas R., Gao Y.T., Dai Q., Yang G., Cai H., Li H., Zheng W., Shu X.O. (2009). Dietary calcium and magnesium intakes and the risk of type 2 diabetes. The Shanghai women’s health study. Am. J. Clin. Nutr..

[10] Liu S., Choi H.K., Ford E., Song Y., Klevak A., Buring J., Manson J.E. (2006). A prospective study of dairy intake and the risk of type 2 diabetes in women. Diabetes Care.

[11] Carson G.R., Edwards N.M. (2009). Chapter 4: Criteria of wheat and flour quality. WHEAT: Chemistry and Technology.

[12] Oda S., Schofield J.D. (1997). Characterisation of friabilin polypeptides. J. Cereal Sci..

[13] Morris C. (2002). Puroindolines: The molecular genetic basis of wheat grain hardness. Plant Mol. Biol..

[14] Silano V., Pocchiari F., Kasarda D.D. (1973). Physical characterization of *alpha*-amylase inhibitors from wheat. Biochim. Biophys. Acta.

[15] Morimoto T., Miyazaki T., Murayama R., Kodama T., Kitamura I., Inoue S. (1999). Wheat album with amylase-inhibitory activity suppresses glycemic rise after rice loading in Human subjects. Jpn. Soc. Nutr. Food Sci..

[16] Piironen V., Lampi A.M., Ekholm P. (2009). Chapter 7: Micronutrients and phytochemicals in wheat grain. WHEAT: Chemistry and Technology.

[17] Kim J., Kweon M. (2024). Quality and noodle-making performance of wheat flour with varied gluten strengths altered by addition of various arabinoxylans. J. Food Sci..

[18] Cormick G., Romero I.B., Puchulu M.B., Perez S.M., Sosa M., Garitta L., Elizagoyen E., Gugole M.F., Belizán J.M., Matamoros N. (2024). A simulation study to improve calcium intake through wheat flour fortification. Public Health Nutr..

[19] Palacios C., Hofmeyr G.J., Cormick G., Garcia-Casal M.N., Peña-Rosas J.P., Betrán A.P. (2021). Current calcium fortification experiences. Ann. N. Y. Acad. Sci..

[20] Michel S., Bayram M. (2024). Kinetics of chemical and color changes in wheat and water during atmospheric cooking as affected by the acidity, hardness, and iron content of water. J. Food Sci..

[21] Bronder K.L., Zimmerman S.L., van den Wijngaart A., Codling K., Johns K.A.G., Pachón H. (2017). Instant noodles made with fortified wheat flour to improve micronutrient intake in Asia: A review of simulation, nutrient retention and sensory studies. Asia Pac. J. Clin. Nutr..

[22] Kanadhia K.C., Shri Ramavataram D.V.S.S., Nilakhe S.P.D., Patel S. (2014). A study of water hardness and the prevalence of hypomagnesaemia and hypocalcaemia in healthy subjects of Surat district (Gujarat). Magnes Res..

[23] Haring B.S., Van Delft W. (1981). Changes in the mineral composition of food as a result of cooking in "hard" and "soft" waters. Arch. Env. Health.

[24] Lee C.-L. (2015). The advantages of deep ocean water for the development of functional fermentation food. Appl. Microbiol. Biotechnol..

[25] Shih M.K., Hsu Q.Y., Liou B.K., Peng Y.H., Hou C.Y. (2020). Deep Ocean Water Concentrate Changes Physicochemical Characteristics, the Profile of Volatile Components and Consumer Acceptance for Taiwanese Rice Shochu. Foods.

[26] Martínez-Martín I., Hernández-Jiménez M., Revilla I., Vivar-Quintana A.M. (2023). Prediction of Mineral Composition in Wheat Flours Fortified with Lentil Flour Using NIR Technology. Sensors.

[27] Zhao J., Na Guo X., Zhu K.X. (2024). Effect of phytic acid on the appearance of yellow alkaline noodles: Color and dark spots. J. Cereal Sci..

[28] Chen M., Wang L., Qian H., Zhang H., Li Y., Wu G., Qi X. (2019). The effects of phosphate salts on the pasting, mixing and noodle-making performance of wheat flour. Food Chem..

[29] Pittas A.G., Dawson-Hughes B., Li T., Dam R.M., Willett W.C. (2006). Vitamin D and calcium intake in relation to type 2 diabetes in women. Diabetes Care.

[30] Yamanaka C., Aoe S. (2018). The effects of dietary calcium levels on pancreatic function in KK Mice. J. Jpn. Soc. Nutr. Food Sci..

[31] Nakamura S., Hasegawa M., Kobayashi Y., Komata C., Katsura J., Maruyama Y., Ohtsubo K. (2022). Palatability and bio-functionality of chalky grains generated by high-temperature ripening and development of formulae for estimating the degree of damage using a rapid visco analyzer of Japonica unpolished rice. Foods.

[32] Nakamura S., Ohtsubo K. (2023). Effects of hard water boiling on chalky rice in term of texture improvement and Ca fortification. Foods.

[33] Kömürcü T.C. (2023). Usage of primitive wheat (*Triticum monococcum* and *Triticum dicoccum*) flour and whole egg in noodle production. Food Sci. Technol. Int..

[34] Zi Y., Cheng D., Li H., Guo J., Ju W., Wang C., Humphreys D.G., Liu A., Cao X., Liu C. (2022). Effects of the different waxy proteins on starch biosynthesis, starch physicochemical properties and Chinese noodle quality in wheat. Mol. Breed..

[35] Inokuma T., Vrinten P., Shimbata T., Sunohara A., Fujita M., Nakamura K., Ishikawa N., Takata K., Kiribuchi-Otobe C., Nakamura T. (2021). Longer Bread Shelf-Life and Improved Noodle-Making Properties Imparted by a Novel Wheat Genotype Are Stable in Different Genetic Backgrounds. J. Agric. Food Chem..

[36] Hai-Teng Li H.T., Li Z., Fox G.P., Gidley M.J., Dhital S. (2021). Protein-starch matrix plays a key role in enzymic digestion of high-amylose wheat noodle. Food Chem..

[37] Nagao S., Imai S., Sato T., Kaneko Y., Otsubo H. (1976). Quality characteristics of soft wheats and their use in Japan. I. Methods of assessing wheat suitability for Japanese products. Cereal Chem..

[38] Toyokawa H., Rubenthaler G.L., Powers J.R., Schanus E.G. (1989). Japanese noodle qualities. I. Flour components. Cereal Chem..

[39] Pulliainen T.K., Wallin H.C. (1994). Determination of total phosphorus in foods by colorimetric measurement of phosphorus as molybdenum blue after dry-ashing: NMKL interlaboratory study. J. AOAC Int..

[40] Yamamoto K., Sawada S., Onogaki I. (1981). Effects of quality and quantity of alkali solution on the properties of rice starch. Denpun. Kagaku..

[41] Juliano B.O., Onate L.M., Mundo A.M. (1965). A simplified assay for milled rice amylose. Food Technol..

[42] Nakamura S., Satoh H., Ohtsubo K. (2015). Development of formulae for estimating amylose content, amylopectin chain length distribution, and resistant starch content based on the iodine absorption curve of rice starch. Biosci. Biotechnol. Biochem..

[43] Toyoshima H., Okadome H., Ohtsubo K., Suto M., Horisue N., Inatsu O., Narizuka A., Aizaki M., Inouchi N., Fuwa H. (1997). Cooperative test on the small-scale rapid method for the gelatinization properties test of rice flours with a rapid visco analyser. Nippon. Shokuhin Kogakukaishi.

[44] Nakamura S., Katsura J., Kato K., Ohtsubo K. (2016). Development of formulae for estimating amylose content and resistant starch content based on the pasting properties measured by RVA of *Japonica* polished rice and starch. Biosci. Biotechnol. Biochem..

[45] Okadome H., Toyoshima H., Sudo M., Ando I., Numaguchi K., Ohtsubo K. (1998). Palatability evaluation for *Japonica* rice grains based on multiple physical measurements of individual cooked rice grain. J. Jpn. Soc. Food Sci. Technol..

[46] McCleary B.M., Monaghan D.A. (2002). Measurement of resistant starch. J. AOAC Int..

[47] Nakamura S., Satoh H., Ohtsubo K. (2011). Characteristics of pregelatinized *ae* mutant rice flours prepared by boiling after preroasting. J. Agric. Food Chem..

[48] Nagao S. (2006). Wheat Science.

[49] Piironen V., Lampi A.M., Ekholm P., Salmenkallio-Marttila M., Liukkonen K.H. (2009). Chapter 7. Minerals and trace elements. WHEAT: Chemistry and Technology.

[50] Balint A.F., Kovacs G., Erdei L., Sutka J. (2001). Comparison of the Cu, Zn, Fe, Ca and Mg contents of the grains of wild, ancient and cultivated wheat species. Cereal Res. Commun..

[51] Akman Z., Kara B. (2003). Genotypic variations for mineral content at different growth stages in wheat (*Triticum aestivum* L.). Cereal Res. Commun..

[52] Blennow A., Nielsen T.H., Baunsgaard L., Mikkelsen R., Engelsen S.B. (2002). Starch phosphorylation: A new front line in starch research. Trends Plant Sci..

[53] Hizukuri S., Takeda Y., Matsubayashi T. (1979). The effect of phosphorus in starch granules on raw starch digestion by bacterial alpha-amylase. J. Jpn. Soc. Starch Sci..

[54] Borkowska-Burnecka J., Leśniewicz A., Zyrnicki W. (2006). Comparison of pneumatic and ultrasonic nebulizations in inductively coupled plasma atomic emission spectrometry–matrix effects and plasma parameters. Spectrochim. Acta B.

[55] Zeng M., Morris C.F., Batey I.L., Wrigley C.W. (1997). Sources of variation for starch gelatinization, pasting, and gelation properties in wheat. Cereal Chem..

[56] Medcalf D.G., Gilles K.A. (1965). Wheat starches 1. Comparison of physicochemical properties. Cereal Chem..

[57] Inouchi N., Hibiu H., Horibata T., Fuwa H., Itami T. (2005). Structure and properties of endosperm starches from cultivated rice of Asia and other countries. J. Appl. Glycosci..

[58] Hirano H., Sano Y. (1998). Enhancement of Wx gene expression and the accumulation of amylose in response to cool temperature during seed development in rice. Plant Cell Physiol..

[59] Yamamori M., Fufita S., Hayakawa K., Matsuki J., Yasui T. (2000). Genetic elimination of a starch granule protein, SGP-1, of wheat generates an altered starch with apparent high amylose. T. Heor. Appl. Genet..

[60] Takeda Y., Hizukuri S., Juliano B.O. (1987). Structures of rice amylopectins with low and high affinities for iodine. Carbohydr. Res..

[61] Duffus C.M., Murdoch S.M. (1979). Variation in starch granule size distribution and amylose content during wheat endosperm development. Cereal Chem..

[62] Regina A., Bird A., Topping D., Bowden S., Freeman J., Barsby T., Kosar-Hashemi B., Li Z., Rahman S., Morell M. (2006). High-amylose wheat generated by RNA interference improves indices of large-bowel health in rats. Proc. Natl. Acad. Sci. USA.

[63] Igarashi T., Yanagihara T., Kanda H., Kawamoto K., Masaki K. (2009). Development of new eating quality evaluation method based on iodine adsorption multispectral analysis of rice flour. J. Crop Sci..

[64] Nakamura S., Yamaguchi H., Benitani Y., Ohtsubo K. (2020). Development of a novel formula for estimating the amylose content of starch using Japonica milled rice flours based on the iodine absorption curve. Biosci. Biotechnol. Biochem..

[65] Appels R. (1998). Insights into noodle quality attributes in wheat. Chem. Aust..

[66] Nakamura S., Katsura J., Maruyama Y., Ohtsubo K. (2018). Relationship between fatty acid composition and starch properties of 30 *japonica* rice cultivars. Cereal Chem..

[67] Takeda T., Hizukuri S. (1982). Location of phosphate groups in potato amylopectin. Carbohydr. Res..

[68] Wren J.J., Merryfield D.S. (1970). Firmly-bound’ lysolecithin of wheat starch. J. Sci. Food Agric..

[69] Fujino Y. (1972). Complex Lipid in Food. Food Hyg. Saf. Sci..

[70] Kim H.O., Hill R.D. (1984). Physical characteristics of wheat starch granule gelatinization in the presence of cyclohepta-amylose. Cereal Chem..

[71] Lin Jane J. (2006). Current understanding on starch granule structures. J. Appl. Glycosci..

[72] Kaneko K., Ota K., Sumino T., Maeda Y. (1989). Effect of anions on binding between calcium and pectic substance. J. Nutr. Sci. Vitaminol..

[73] Kainuma K., Yamamoto K., Suzuki S., Takaya K., Fuwa H. (1978). Studies on structure and physico-chemical properties of starch. Part IV. Structural, chemical and rheological properties of air classified small-and large granule potato starch. J. Jpn. Soc. Starch Sci..

[74] Kainuma K., Miyamoto S., Yoshioka S., Suzuki S. (1976). Studies on structure and physico-chemical properties of starch. J. Jpn. Soc. Starch Sci..

[75] Nakamura S., Satoh A., Aizawa M., Ohtsubo K. (2022). Characteristics of physicochemical properties of chalky grains of *Japonica* rice generated by high temperature during ripening. Foods.

[76] Nakamura S., Katsura J., Maruyama Y., Ohtsubo K. (2021). Evaluation of hardness and retrogradation of cooked rice based on its pasting properties using a novel RVA testing. Foods.

[77] Nakamura S., Katsura J., Suda A., Maruyama Y., Ohtsubo K. (2024). Effects of binding between Ca in hard water and phosphorus in amylopectin on the qualities of boiled rice and rice noodles prepared by soaking and boiling in hard water. Foods.

[78] Fukaya M., Takasu A., Yamada E., Tsukamoto Y., Furukawa Y. (1998). Characterization of solubilization of insoluble calcium and magnesium with various kinds of vinegar. Nippon Shokuhin Kagaku Kogakukaishi.

[79] Southgate D.A.T. (1987). Minerals trace elements, and potential hazards. Am. J. Clin. Nutr..

[80] Yanaka M., Takata K., Funatsuki W., Ishikawa N., Takahashi T. (2017). Effects of the composition of glutenin subunits controlled by the *Glu-A1* and *Glu-D1* and protein content on the noodle quality in Japanese soft wheat. Jpn. J. Crop Sci..

[81] Oda M., Yasuda Y., Okazaki S., Yamauchi Y., Yokohama Y. (1980). A method of flour quality assessment for Japanese noodles. Cereal Chem..

[82] Crosbie G.B., Lambe W.J., Tsutsui H., Gilmour R.F. (1992). Further evaluation of the flour swelling volume test for identifying wheats potentially suitable for Japanese noodles. J. Cereal Sci..

[83] Crosbie G.B. (1991). The relationship between starch swelling properties, paste viscosity and boiled noodle quality in wheat flours. J. Cereal Sci..

[84] Noda T. (2021). The preparation and food applications of divalent cation-substituted potato starch. J. Biorheol..

[85] Noda T., Takigawa S., Matsuura-Endo C., Ishiguro K., Nagasawa K., Jinno M. (2015). Properties of Calcium-fortified potato starch prepared by immersion in natural mineral water and its food application. J. Appl. Glycosci..

[86] KeeBaik B., Czuchajowska Z., Pomeranz Y. (1995). Discoloration of dough for oriental noodles. Cereal Chem..

[87] Paznocht L., Kotikova Z., Orsak M., Lachman J. (2019). Carotenoid changes of colored-grain wheat flours during bun-making. Food Chem..

[88] Hou G. (2001). Oriental noodles. Adv. Food Nutr..

[89] Ito M., Ohta K., Nishio Z., Tabiki T., Hashimoto N., Funatsuki W., Miura H., Yamauchi H. (2007). Quality evaluation of yellow alkaline noodles made from the KItanokaori wheat cultivar. Food Sci. Technol. Res..

[90] Sajilate M.G., Singhal R.S., Kulkarni P.R. (2006). Resistant starch: A review. Compr. Rev. Food Sci. Food Saf..

[91] Nagahata Y., Kobayashi I., Goto M., Nakaura Y., Inouchi N. (2013). The formation of resistant starch during acid hydrolysis of high-amylose corn starch. J. Appl. Glycosci..

[92] Shimada R., Yoshimura M. (2021). Effect of resistant starch type 4 from different starch sources on the physical properties and palatability of bread. J. Biorheol..

[93] Tabiki T., Takata K., Nishio Z., Kuwabara T., Ozeki S., Tabaya S., Iriki N., Yamauchi H., Ichinose Y. (2006). “Kitanokaori”: A new winter wheat variety. Res. Bull. Nalt. Agric. Res. Cent. Hokkaido Reg..

[94] Rouau X., Moreau D. (1993). Modification of some physicochemical properties of wheat flour pentosans by an enzyme complex recommended for baking. Cereal Chem..

[95] Michniewicz J., Biliaderis G.G., Bushuk W. (2001). Effect of added pentosans on some properties of wheat bread. Food Res. Technol..

[96] Hashimoto S., Hino A., Yamaguchi Y., Kai T. (1999). Isolation and characterization of pentosans from some species of wheat flour. Bull. Nakamura Gakuen Univ..

[97] Shogren M.D., Hashimoto S., Pomeranz Y. (1987). Cereal pentosans: Their estimation and significance. II. Pentosans and breadmaking characteristics of hard red winter wheat flours. Cereal Chem..

[98] Shibuya N., Misaki A. (1978). Structure of hemicellulose isolated from rice endosperm cell wall: Mode of linkages and sequences in xyloglucan, β-glucan and arabinoxylan. Agric. Biol. Chem..

[99] Ogata M., Iwasaki N., Uchigata Y. (2017). Osteoporosis as a Complication of Diabetes.

[100] Farlay D., Armas L.A.G., Gineys E., Akhter M.P., Recker R.R., Boivin G. (2016). Nonenzymatic glycation and degree of mineralization are higher in bone from fractured patients with type 1 diabetes mellitus. J. Bone Miner Res..

[101] Oei L., Zillikens M.C., Dehghan A., Buitendijk G.H.S., Castano-Betancourt M.C., Estrada K., Stolk L., Oei E.H.G., Meurs J.B.J., Janssen J.A.M.J.L. (2013). High bone mineral density and fracture risk in type 2 diabetes as skeletal comolications of inadequate glucose control: The Rotterdam Study. Diabetes Care.

