# Supplementary material for: Characteristics of Wheat Noodle “Kitanokaori” Using Weakly Acidic Hard Water in Terms of Functional Qualities, Such as Inhibiting Postprandial Abrupt Increase in Blood Glucose
Source: Foods. 2025 Mar 19;14(6):1044. doi: 10.3390/foods14061044 (PMC11941889; doi:10.3390/foods14061044)
Supplement: Supplementary file 1 [file foods-14-01044-s001.zip › foods-3514161-supplementary figures.pdf]

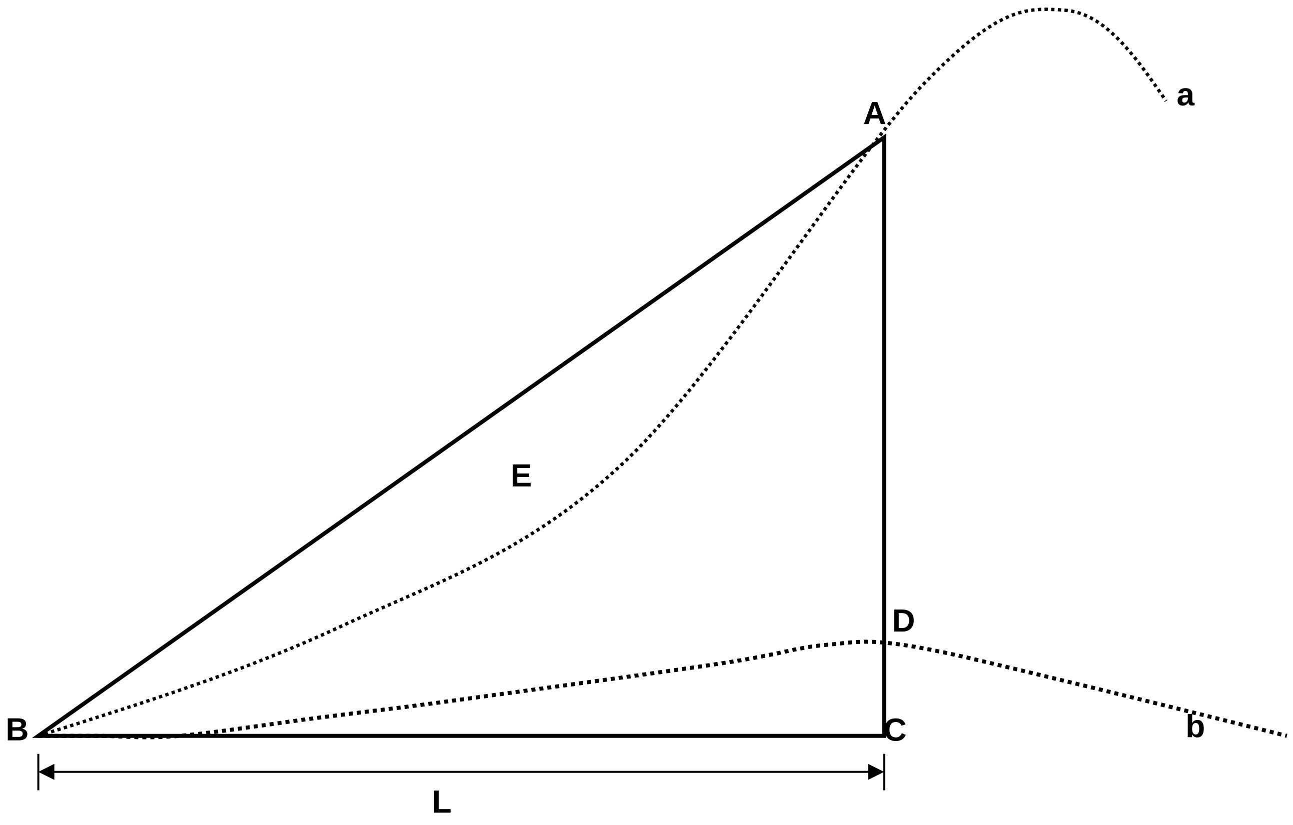

a : The compression force curve

b : Back pressure force curve

Toughness : area of AEBC(curve surface)
Pliability : area of ABC / area of AEBC
Brittleness : grain thickness / L
Tenderness : Elastic limit compression force (A)
Max.Length : Elastic limit length (L)

Continuous progressive compression test (CPC test)

Figure S1.

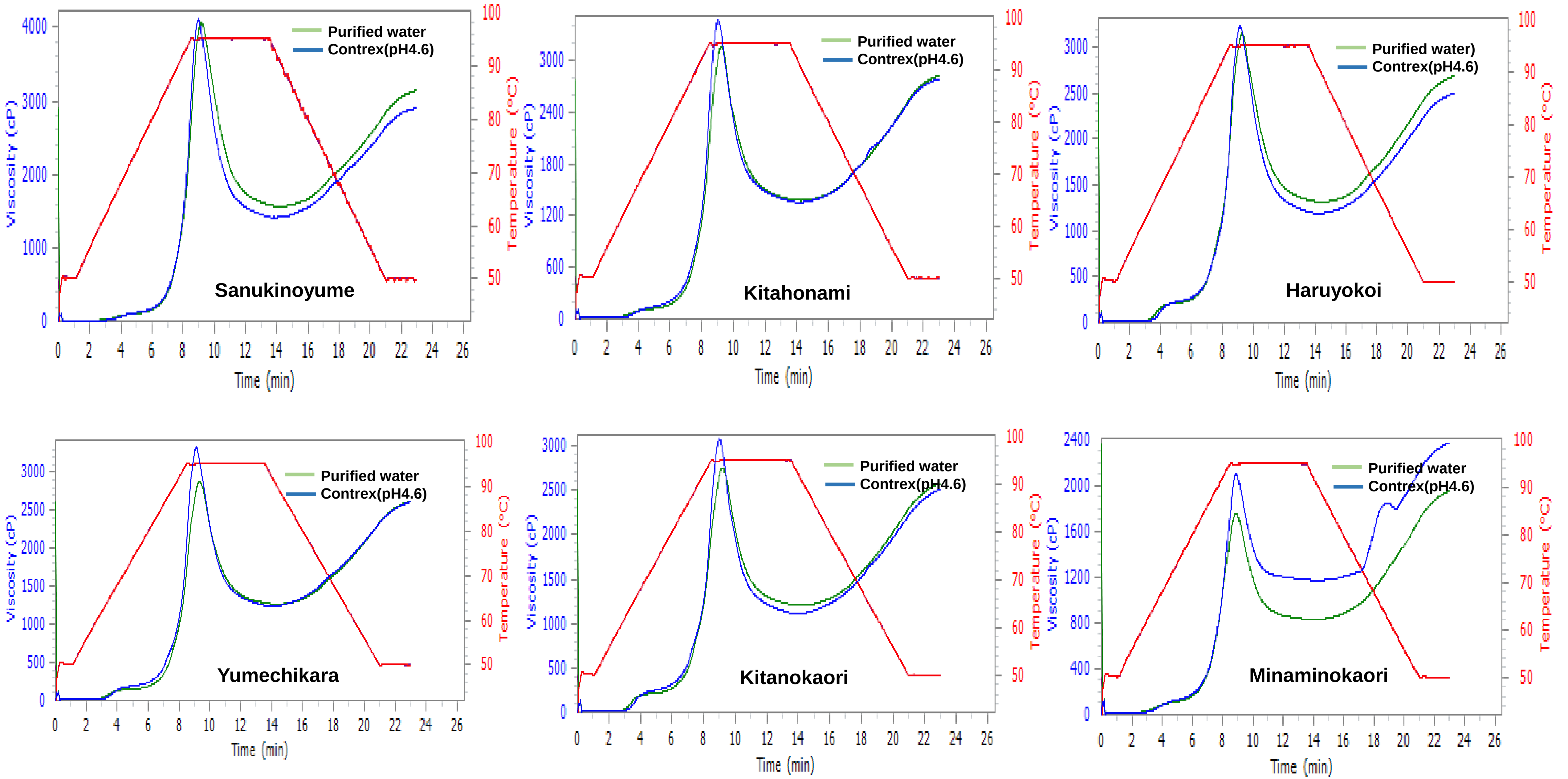
Sanukinoyume
Kitahonami
Haruyokoi
Yumechikara
Kitanokaori
Minaminokaori
Purified water
Contrex(pH4.6)
Purified water)
Viscosity (cP)
Temperature (°C)
Time (min)

Figure S2.
